# Liposomal DQ in Combination with Copper Inhibits ARID1A Mutant Ovarian Cancer Growth

**DOI:** 10.3390/biom13050744

**Published:** 2023-04-25

**Authors:** Xuejia Kang, Qi Wang, Siqi Wu, Chuanyu Wang, Manjusha Annaji, Chung-Hui Huang, Jianzhong Shen, Pengyu Chen, R. Jayachandra Babu

**Affiliations:** 1 Department of Drug Discovery and Development, Harrison College of Pharmacy, Auburn University, Auburn, AL 36849, USA; 2 Materials Research and Education Center, Materials Engineering, Department of Mechanical Engineering, Auburn University, Auburn, AL 36849, USA

**Keywords:** ovarian clear cell cancer (OCCC), liposome, ROS-responsive prodrug, glutathione (GSH), epithelial-mesenchymal transition (EMT), immunogenic cell death (ICD)

## Abstract

Therapeutic strategies for ARID1A-mutant ovarian cancers are limited. Higher basal reactive oxygen species (ROS) and lower basal glutathione (GSH) empower the aggressive proliferation ability and strong metastatic property of OCCCs, indicated by the increased marker of epithelial-mesenchymal transition (EMT) and serving the immunosuppressive microenvironment. However, the aberrant redox homeostasis also empowers the sensitivity of DQ-Lipo/Cu in a mutant cell line. DQ, a carbamodithioic acid derivative, generates dithiocarbamate (DDC) in response to ROS, and the chelation of Cu and DDC further generates ROS and provides a ROS cascade. Besides, quinone methide (QM) released by DQ targets the vulnerability of GSH; this effect, plus the increase of ROS, destroys the redox homeostasis and causes cancer cell death. Also importantly, the formed Cu(DDC)_2_ is a potent cytotoxic anti-cancer drug that successfully induces immunogenic cell death (ICD). The synergistic effect of EMT regulation and ICD will contribute to managing cancer metastasis and possible drug resistance. In summary, our DQ-Lipo/Cu shows promising inhibitory effects in cancer proliferation, EMT markers, and “heat” the immune response.

## 1. Introduction

Epithelial ovarian carcinoma (EOC) is one of the most common malignancies worldwide [1,2]. It is a heterogeneous group of diseases with distinct histologic subtypes, including mucinous, low-grade, and high-grade serous, endometrioid, and clear cells [2]. Among them, ovarian clear cell carcinoma (OCCC) is the second most common subtype after high-grade serous carcinoma (HGSC), accounting for 10% of all EOCs in North America [3]. Moreover, because of the refractory property of standard platinum-based chemotherapy, OCCC is a relatively aggressive type [4].

Elucidating the molecular basis of OCCC is crucial to developing targeted therapy [5]. Recently, cancer genome sequencing has identified genetic factors such as gene mutation and aberrant chromatin regulation playing a major role in OCCC tumorigenesis and progression [6]. The switch/sucrose non-fermentable (SWI/SNF) chromatin-remodeling complex mobilizes nucleosomes and regulates gene expression as well as chromatin dynamics [7,8]. The inactivation of subunits of SWI/SNF is a frequent phenomenon in a variety of cancer types [8]. AT-rich interactive domain-containing protein 1A (ARID1A), which encodes a component of the SWI/SNF chromatin-remodeling complex, is mutated in ∼57% of ovarian clear cell carcinomas (OCCCs) [7,8].

ARID1A is a tumor suppressive factor. Patients with decreased ARID1A have a poor prognosis, and the mutation of ARID1A promotes metastases of cancers [8]. Though the mutant OCCC was discovered recently [8], the metastatic mechanism of ARID1A mutant ovarian clear cell cline and therapeutic strategies for ARID1A-mutant cancers remain less reported; thus, associated investigations are needed.

ARID1A mutant cancer cells showed an elevated basal level of reactive oxygen species (ROS) owing to aberrant metabolism [9]. Meanwhile, the aberrant level of glutathione GSH has been unfolded. It is rational to hypothesize that there is a new redox homeostasis in the ARID1A mutant cell line and that breaking this balance will contribute to the inhibition of mutant OCCC proliferation. In addition, the higher ROS benefits the drug resistance of cancer cells associated with epithelial-mesenchymal transition (EMT), related to metastasis and drug resistance. Thus, there is an association between the presence or absence of ARID1A and the regulation of EMT, though it remains unknown. To our knowledge, the research of ARID1A on biomarkers of EMT is limited. Thus, this investigation is significant for improving the therapeutics of OCCCs.

When considering the candidate for ARID1A OCCC treatment, besides an increase in ROS levels that can promote cell proliferation and EMT, it is essential to consider other factors as well [10]; excessive ROS induced by agents that cause oxidative stress results in cell death [10,11]. As mentioned, if we could break the new redox balance in ARID1A mutant cancer cells, therapeutic effects could be achieved. DQ is a disulfiram-related prodrug responsive to reactive oxygen species (ROS) with potent anti-cancer effects [12,13]. Nevertheless, the use of DQ may be limited by the hydrophobic nature of the drug; proper delivery of the therapeutics will benefit DQ use in cancer therapy. Biocompatible and biodegradable coating material ensures the safety and stability of the delivery system [14,15,16]. Liposomes have been used for clinical application, and recently, they were used for vaccine delivery and immunotherapy [17,18]. The findings for liposomes for hydrophilic drugs, such as miRNA delivery, matter to researchers, especially during the pandemic [19,20]. In addition, the liposome lipid bilayer empowers its ability to cover hydrophobic drugs [21]. In this regard, the encapsulation of DQ within liposomes will be beneficial to resolving the concern of DQ’s poor solubility.

**Scheme 1:**
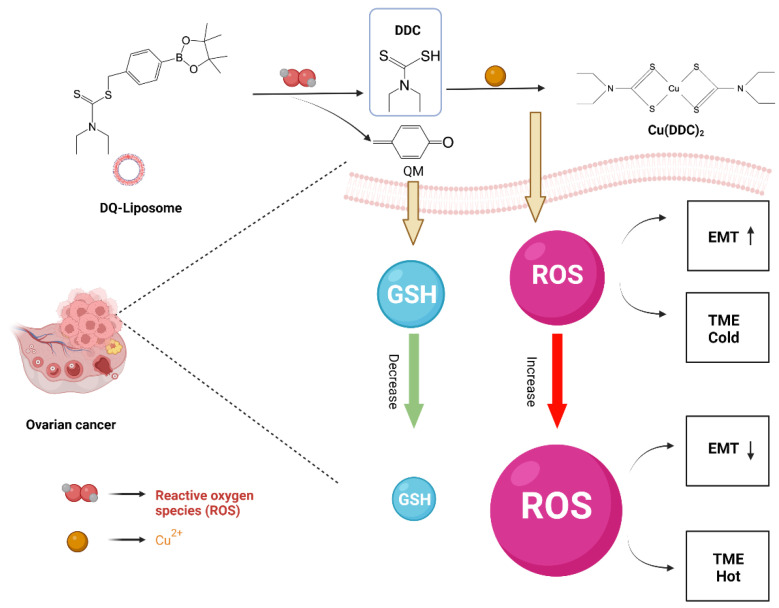
The anti-cancer mechanism of DQ-Lipo/Cu. Several mechanisms are possible for anti-cancer activity: Firstly, active anti-cancer metabolite was produced from DQ under the ROS activation, and DDC chelates the available Cu(II) from CuCl_2_ to give the cytotoxic drug Cu(DDC)_2_. Secondly, another metabolite, QM, consumes the GSH. In this case, oxidative stress amplification is achieved and exerts anti-cancer activity. Thirdly, the ROS increase and resultant decrease of GSH contribute to the complete redox balance and damage to cancer cell proliferation. The additive effects of the system exert potency in inhibiting ARID1A mutant cancer. The up arrow indicates the EMT performce increase, the down arrow suggestes the EMT performace decrease (Created in biorender.com, accessed on 12 March 2023).

So, in this study, we developed a liposomal delivery system for DQ (termed as DQ-Lipo), then its antitumor efficacy in combination with CuCl_2_ was investigated. Serval mechanisms were involved in this strategy (Scheme 1). First, the entrapped DQ was released from the liposomes; it generated dithiocarbamate (DDC) triggered by cellular ROS; meanwhile, the feedback loop occurred as the chelation process of DDC and Cu produced ROS and further increased the metabolism of DQ. Secondly, quinone methide (QM) release showed effects in consuming the GSH and further increased ROS levels. Ultimately, the high ROS increased, and GSH became more vulnerable, resulting in the complete collapse of redox homeostasis in cancer cells and causing cell death. Finally, the released DDC could complex with Cu, forming Cu(DDC)_2_, which showed promising anti-cancer potential and triggered immunogenic cancer cell death (ICD). With these synergistic mechanisms, this system could effectively inhibit cancer cell proliferation and contribute to treating ARID1A mutant OCCCs.

## 2. Materials and Methods

### 2.1. Materials

Soybean phosphatidylcholine (SPC), cholesterol, and DSPE-PEG2000 were purchased from Avanti lipids (Avanti Polar Lipids, Alabaster, AL, USA). Dimethyl sulfoxide (DMSO), copper chloride (CuCl_2_), and 3-(4,5-Dimethylthiazol-2-yl)-2,5-diphenyltetrazolium bromide (MTT) were purchased from Sigma-Aldrich (St. Louis, MO, USA). Fetal bovine serum (FBS) and DMEM-F12 medium (access date 12 March 2021,Gibco, Grand Island, NY, USA) were purchased from Thermo Fisher Scientific (Waltham, MA, USA), respectively. DQ was synthesized in our laboratory based on a previous report [12].

### 2.2. Cell Lines

We performed the studies using ARID1A wildtype OCCC RMG1 cells with or without shRNA-mediated ARID1A knockdown and termed as ARID1A-Mutant (ARID1A-MT) and ARID1A-Wildtype (ARID1A-WT) (Wistar Institute, Philadelphia, PA, USA). The cells were cultured in 1:1 Dulbecco’s modified Eagle’s medium (DMEM): F12 supplemented with 10% FBS and antibiotics (100 µg/mL streptomycin and 100 µg/mL penicillin) at 37 °C and 5% CO_2_ [22].

### 2.3. Preparation of Liposomes

We synthesized DQ with 4-Bromomethylphenylboronic acid pinacol ester and sodium diethyldithiocarbamate [12]. The liposomes were prepared using a thin-film hydration technique. Lipids (SPC/Chol/DSPE-PEG2000) at a molar ratio of 39:8:0.7 were dissolved in Dichloromethane (DCM) and then mixed with DQ in DCM solution. The ratio between DQ and lipids was 1:10 (*w*/*w*). The organic solvents were removed via a rotary vacuum evaporator in a 45 °C water bath. The lipid film thus formed was hydrated with water. The suspensions were dispersed and extruded through a polycarbonate membrane (200 nm) using an extruder (Avanti Polar Lipids, Alabaster, AL, USA). The unencapsulated drug was removed using a dialysis bag (3.5 KDa molecular weight). The liposomes were termed as DQ-Lipo.

### 2.4. Characterization of Liposomes

The DQ concentration in micelle formulations was determined using a UV–Vis spectrophotometer at 270 nm. The drug loading (DL%) and encapsulation efficiency (EE%) of the liposomes were calculated by the equations:

DL% = WDE/WL × 100%; EE% = WDE/WT × 100%.

where WDE was the drug amount encapsulated in liposomes, WL was the total amount of the DQ and polymer carriers, and WT was the total amount of the drug.

The morphology was determined via transmission electron microscopy (TEM), scanning electron microscopy (SEM) (Zeiss EM10), and atomic force microscopy (AFM). The size and charge of liposomes were determined with DLS (Zetasizer, Malvern Instruments Ltd., Worcestershire, UK). To determine the surface charge, the zeta potential of the liposomal formulations was measured on a Nanosizer (Malvern Instruments Ltd., UK) with a 1-mL cuvette. Each measurement was performed in triplicate.

The serum stability of DQ-Lipo was determined by measuring the change of particle size in FBS (10%) containing phosphate-buffered saline (PBS) (pH 7.4). The long-term stability of DQ-Lipo was determined by measuring the change of particle size in PBS (pH 7.4) at 4 °C.

The in vitro drug release was conducted using a dialysis method [23]. The liposomes were placed in a pre-wetted dialysis tube with a cellulose ester membrane with a molecular weight cutoff of 14 KDa. To get the release profile of DQ-Lipo, the dialyzed solution was a PBS/Tween 80 mixture with pH 7.4, and the release assay was conducted in a 37 °C incubator shaker (110 rpm). Finally, drug concentrations were measured at varying time points. The experiments were performed in triplicate.

### 2.5. In Vitro Cell Assays

The cell viability was measured following the previous MTT method [21,24]. For the sensitivity assay of DQ-Lipo/Cu in the ARID1A-WT and ARID1A-MT cell lines, both cell lines were plated into the 96-well plates at 1 × 10^4^ cells per well. When they reached 70% confluency, fixed CuCl_2_ and the DQ-Lipo at the molar ratio of 1:4 (optimized for the chelation of DQ-Lipo/Cu) and DQ-Lipo at a series of concentrations were diluted and added. The MTT reagent was added after 48 h drug treatments on the incubated cells in the culture medium. The absorption value marked as OD was measured with a reader. The cell viability was calculated based on the formulation:

Cell viability (%) = ODtest/(ODcontrol) × 100%.

To learn the drug effects in 2D and 3D cell models, we also established the 3D tumor sphere model. Briefly, single cell suspension at a density of 1 × 10^3^/well was plated in a 96-well plate covered with Matrigel based on the published protocol. The fresh medium changed every two days and waited for 10 days and media with a series concentration of drugs (CuCl_2_:DQ at a molar ratio of 1:4 for 48 h). At the end of treatments, the alamar blue as an indicator for cell viability solution was added, and the fluorescence intensity was measured at EX = 540 nm and EM = 590 nm.

To understand the advantages of the drug combination in the ARID1A-MT cell line, we seeded the mutant cell line in a 96-well plate and then set the groups as I Control group, II vehicle, III, CuCl_2_ (0.5 μM) IV DQ-Lipo (2 μM), V. DQ-Lipo (2 μM)/CuCl_2_ (0.5 μM); after 48 h treatment, MTT reagent was added for 4 h, and 200 μL DMSO was used to dissolve the purple sediment crystal and read the absorbance at 570 nm using a cytation 5 cell imaging multi-mode reader.

For ROS measurement, we used the DCFH-DA as a probe. We incubated the cells with 10 μM DCFH-DA for 1 h and washed them with PBS 2 times, and observed using a cytation 5 cell imaging multi-mode reader.

To test the anti-cancer efficacy of different formulations on ARID1A-MT cell lines using calcein AM PI staining, CuCl_2_ (0.5 μM), DQ-Lipo (2 μM), DQ-Lipo (2 μM)/CuCl_2_ (0.5 μM) were added to the cells and cultured for 48 h. Then the calcein AM and PI at 5 μM concentration were added for 1 h, and the images were taken by a cytation 5 cell imaging multi-mode reader.

### 2.6. EMT Gene Expression and Western Blotting Assay

To determine the gene expression difference in ARID1A-wildtype and mutant cell lines, we determined the expression levels of EMT genes in these two cancer cell lines with real-time qPCR with SYBR Green-based method. qPCR was conducted based on the protocol: For stage 1. Activation: 50 °C for 3 min; For stage 2: pre-soak: 95 °C for 5 min; For stage 3: Denaturation temperature set as 95 °C and run for 15 s, Annealing: 60 °C for 1 min (recycle 40); Stage 4: Melting curve: 95 °C for 15 s, 60 °C for 15 s, 95 °C for 15 s. The RNA was isolated with the E.Z.N.A.^®^ HP Total RNA Kit (omega) [25]. The mRNA is based on a standard approach and normalized with the control (no treatment on cancer cells). Furthermore, the primers are listed in Figure S1.

The expression of ARID1A in mutant and wildtype cell lines was measured using western blotting assay. Briefly, we used the 10% gel, and the running setting was 80 V for 0.4 h plus 120 V for 1.2 h. Then, semi-dry transfer with 0.45 um PVDF membrane was used to transfer the gel (12 V, 1.2 h). Afterwards, the primary anti-ARID1A Antibody (C-7): sc-373784 (Santa Cruz Biotechnology, Dallas, TX, USA) was used to incubate the membrane for 12 h followed by 1 h blocking, the β-Actin (D6A8) Rabbit mAb #8457 was used as the loading reference. We used the secondary antibody to further incubate the membrane after washing the primary antibody. Finally, Pierce™ ECL western blotting substrate (catalog number: 32106, Thermo Fisher Scientific, Waltham, MA, USA) was used to reveal the membrane and get the band.

The expression of ARID1A in mutant and wildtype cell lines was measured using western blotting assay [24]. Briefly, we used the 10% gel, and the running setting was 80 V for 0.4 h plus 120 V for 1.2 h. Then, semi-dry transfer with 0.45 um PVDF membrane was used to transfer the gel (12 V, 1.2 h). Afterwards, the primary anti-ARID1A Antibody (C-7): sc-373784 (Santa Cruz Biotechnology, Dallas, TX, USA) was used to incubate the membrane for 12 h followed by 1 h blocking, the β-Actin (D6A8) Rabbit mAb #8457 was used as the loading reference. We used the secondary antibody to further incubate the membrane after washing the primary antibody. Finally, Pierce™ ECL western blotting substrate (catalog number: 32106, Thermo Fisher Scientific, Waltham, MA, USA) was used to reveal the membrane and get the band.

### 2.7. Cytokines Detection in the Co-Culture System and Biomarkers of ICD

To investigate the roles of the ARID1A- mutant cell line in TME and the impact of DQ-Lipo/Cu in factors in TME, we co-cultured the non-treated and treated cancer cell with the PMA-induced THP-1 monocytes according to published methods [26,27]. Briefly, original THP-1 cells were grown in suspension in RPMI/FCS media; then cells were seeded in 24 well plates at 2 × 10^5^ cells/well. 10 ng/mL of 12-O-tetradecanoylphorbol-l3-acetate (PMA) (Sigma-Aldrich) was used to treat THP-1 for 24 h. Then, we incubated the above differentiated and attached macrophage with cancer cells. Prior to the co-culture, cancer cells were treated with medium, blank liposomes, CuCl_2_ (0.5 μM), DQ-Lipo (2 μM), and DQ-Lipo (2 μM)/CuCl_2_ (0.5 μM), respectively, for 4 h (the short incubation time minimized the toxicity toward monocytes). Then cells were collected and washed twice with PBS; after counting, the same amount of cancer cells (1 × 10^5^ cells/insert) in different groups were seeded in the inserts of the transwell plate (0.4 μm). The bottom chambers were monocytes at a density of 2 × 10^5^ cells/well. After two days of co-culture, we determined the cytokines levels (TNF-α and TGF-β) with ELISA kits (Human TNF-alpha Quantikine ELISA Kit Catalog#: DTA00D, and Human/Mouse/Rat/Porcine/Canine TGF-beta 1 Quantikine ELISA kit Catalog#: DB100C).

For ICD markers, cells were seeded in a 24-well plate (1 × 10^5^ cells per well) and further incubated overnight [12,16]. Then, cells were treated with different formulations for 24 h; the following two ICD biomarkers were analyzed. (1) High mobility group box 1 protein (HMGB1): Cell culture media was collected after treatment, and the HMGB1 in the culture media was determined with an HMGB1 ELISA chemiluminescence kit (Novus Biologicals). (2) ATP release. The ATP concentration in the collected culture media was determined using an ATP bioluminescence detection kit (Promega, Madison, WI, USA).

### 2.8. Statistical Analysis

Data analyses were performed using GraphPad Prism 6 software. All data were depicted as the mean ± SD from ≥3 experiments or samples. The Student’s *t*-test was used when two parameters were evaluated. One-way ANOVA was employed in the difference between three or more groups. * *p* < 0.05, ** *p* < 0.01, *** *p* < 0.001.

## 3. Results and Discussion

### 3.1. Characterization, Stabilities, Drug Entrapment Efficiency, and Drug Release of Liposomes

**Figure 1:**
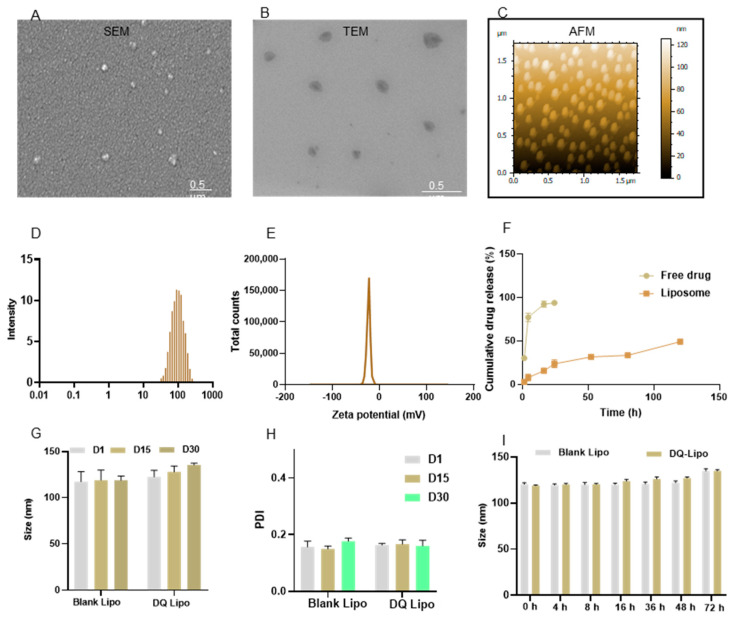
Characterization of the DQ-liposomes for various properties. (**A**) The SEM image of DQ-Lipo. (**B**) The TEM image of DQ-Lipo. (**C**) AFM result of DQ-Lipo. (**D**) The particle size distribution of DQ-Lipo. (**E**) The zeta potential of DQ-Lipo. (**F**) Drug release profile of DQ Lipo. (**G**) Long-term physical stability of blank liposomes and DQ liposomes showing the particle size. (**H**) Long-term physical stability of blank liposomes and DQ liposomes showing the PDI. (**I**) Serum stability of blank liposomes and DQ liposomes for the stable particle size. D1, D15, and D30 represent the stability data collected on 1, 15, and 30 days, respectively. Data are presented as the mean ± SD, *n* = 3.

DQ, a chemically related to DDC, has shown potent activity against breast cancer cell lines [22]. However, owing to the hydrophobic property of the DQ, it poses formulation challenges for its effective intra-tumoral delivery. Nanotechnology via liposomal formulation offers several advantages, such as drug solubilization, drug loading efficiency, biocompatibility, and intracellular delivery in many cancers. Due to the merits and the successful translation of liposomes, it is rational to choose liposomes as carriers for DQ. SEM, TEM, and AFM were used to visualize the morphology of liposomes, and the data is shown in Figure 1A–C. The mean diameters for DQ-Liposomes were 122.5 ± 5.7 nm and showed uniform dispersion (Figure 1D). The zeta potential was −20.6 ± 2.5 mV (Figure 1E), consistent with other studies [28].

The drug release curve shown in Figure 1F indicates the slow-release profiles of liposomes. The mean diameters for blank liposomes were 117.2 ± 8.9 nm and showed stability for 30 days (Figure 1G,H). The particle size and PDI of liposomes did not appreciably change, as shown by the stability data for up to 30 days. The PDI and size of liposomes in a serum-containing medium were monitored over time. The liposomes exhibited a slight increase in size and a small increase in the PDI over a period at 37 °C, and these increases were statistically insignificant (Figure 1I). The successful synthesis of DQ was confirmed by ESI-MS (Figure S2). The EE% of DQ-Lipo was (76.9 ± 3.4)%, and the DL% of DQ-Lipo was (5.9 ± 0.3)%, respectively.

### 3.2. ARID1A-Mutant Cancer Cell Line Is More Sensitive to the DQ-Lipo/Cu

**Figure 2:**
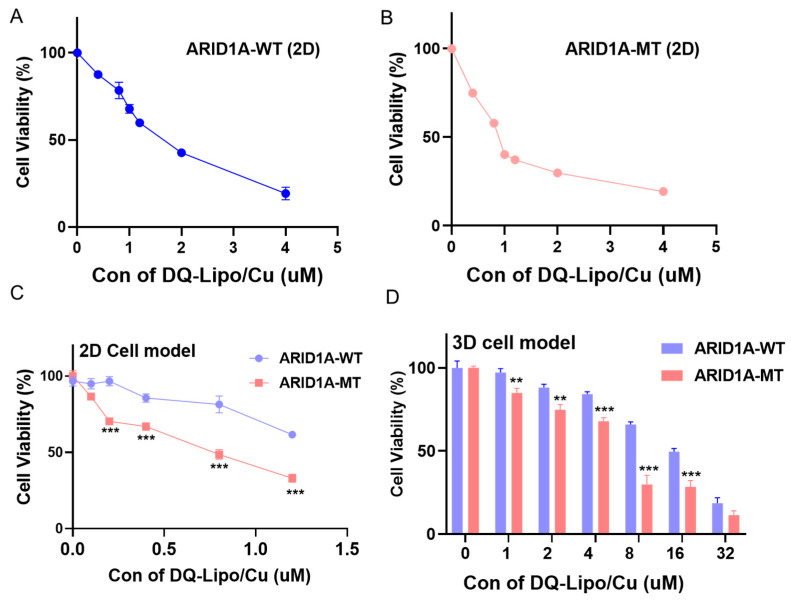
Cell viability in ARID1A wildtype (ARID1A-WT) and ARID1A-mutant (ARID1A-MT) cell lines. (**A**) The anti-cancer efficacy of DQ-Lipo/Cu on ARID1A wildtype (ARID1A-WT) treated with a serial of Cu/DQ concentration for 48 h. (**B**) The anti-cancer efficacy of DQ-Lipo/Cu on ARID1A-mutant (ARID1A-MT) treated with a serial of Cu/DQ concentration for 48 h. (**C**) The difference of cell viability on 2D ARID1A wildtype (ARID1A-WT) and ARID1A-mutant (ARID1A-MT) cell model treated with a series of Cu/DQ concentrations. (**D**) The anti-cancer efficacy of DQ-Lipo/Cu on 3D ARID1A wildtype (ARID1A-WT) and ARID1A mutant (ARID1A-MT) tumor sphere treated with a serial of Cu/DQ concentration for 48 h. The molar ratio of CuCl_2_: DQ is 1:4. Data are presented as the mean ± SD, *n* = 3. ** *p* < 0.01, *** *p* < 0.001, unpaired *t*-test.

To learn the difference in ROS levels and whether the ARID1A-mutant and ARID1A-wildtype cells have a sensitive preference towards DQ-Lipo/Cu, we first detected the basal ROS levels in both cell lines. The ARID1A-mutant cell line had higher ROS basal levels than the ARID1A-wildtype cell (Figure S3). The protein levels of ARID1A were measured using a western blot (Figure S4). Associated S3 with S4A negative correlation between ARID1A expression with ROS levels was found in ovarian clear cancer cells. Increased intracellular ROS levels, along with the absence of tumor suppressor-ARID1A, were the main causes of aggressive cell growth in OCCC [29]. A distinct ratio of ROS/GSH was in the mutant cancer cell, but the cancer cell survival and proliferation showed aggressive properties, such as easier metastasis and platinum resistance [30,31]. Thus, dealing with the aberrant redox balance benefited the management of OCCC [9,29]. The cell viability assay was used in both 2D and 3D models. In Figure 2A,B, we presented the initial cell viability data based on higher concentrations. However, in Figure 2C, we repeated the experiment using various lower concentrations to determine the potency and IC50 values. Even in a relatively lower concentration, the DQ-Lip/Cu showed its potency on the mutant cell lines. DQ-Lipo/Cu demonstrated the greatest difference in cytotoxicity between ARID1A-mutant and ARID1A-wildtype cancer cell lines (Figure 2C). Furthermore, the inhibition of cancer cell growth was concentration-dependent, and it should be noted that the ARID1A-mutant cancer cell lines had significantly lower IC50 of DQ-Lipo/Cu than the ARID1A-normal cancer cell lines. The IC50 of DQ-Lipo/Cu was around 2.5 uM, whereas the DQ-Lipo/Cu on ARID1A-wildtype cancer cells was around 0.78 uM (Figure 2A,B). Furthermore, DQ-Lipo/Cu had much stronger inhibitory effects on the ARID1A-mutant cancer cell sphere (Figure 2D). The cell viability remained at 84.28 ± 1.3% at 4 uM, while the cell viability at 4 uM was 67.99 ± 2.03%, showing a statistical difference (*p* = 0.0003). Similarly, the concentrations at 8 and 16 uM showed statistical significance (*p* = 0.0001 and *p* = 0.0009, respectively (Figure 2C,D). A series of DQ-Lipo/Cu at lower concentrations were also tested using MTT, as shown in Figure 2E, and the curve revealed a significant difference between the ARID1A-WT and ARID1A-MT cell lines. The cell viability of AR-ID1A-WT and ARID1A-MT cell lines was unaffected by different formulations, including the blank liposome and copper group. However, DQ-Lipo and DQ-Lipo/Cu were much more effective in the ARID1A-MT cell line. These findings suggest that the AR-ID1A-mutant cancer cell line is more sensitive to DQ-Lipo/Cu.

### 3.3. ARID1A-Mutant Cancer Cell Line Shown Different EMT Biomarkers Levels

**Figure 3:**
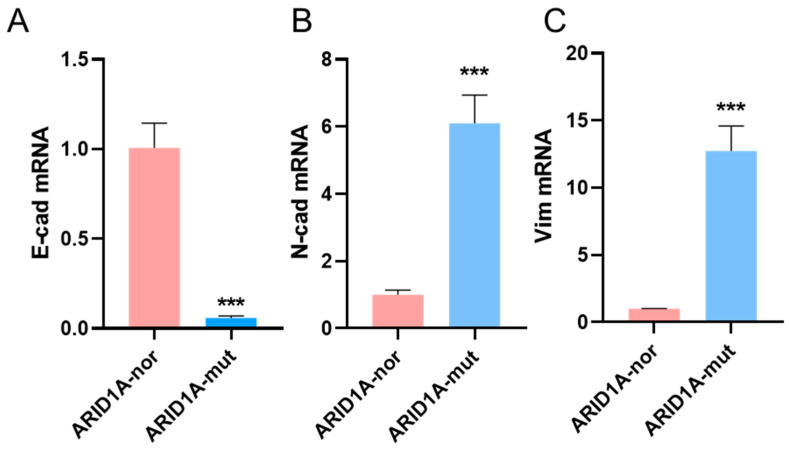
(**A**) Expressions of E-cadherin in ARID1A wildtype (ARID1A-WT) and ARID1A-mutant (ARID1A-MT) cell lines. (**B**) The expressions of N-cadherin in ARID1A-WT and ARID1A-MT cell lines (**C**). The expressions of Vimentin in ARID1A-WT and ARID1A-MT cell lines Vimentin. (Data are presented as the mean ± SD, *n* = 3. ***, *p* < 0.001, unpaired *t*-test).

The downregulation of ARID1A was confirmed by western blot (Figure S4). It was reported that ARID1A silencing promoted migration, invasion, and angiogenesis [31]. EMT is critical during cancer invasion and metastasis [32]. Earlier studies reported that ARID1A-mutant cancer cells show enhanced invasiveness associated with the EMT in cancer cells [33]. For instance, ARID1A downregulation promoted colorectal cancer (CRC) metastasis via E-cadherin [34,35,36]. So, we checked EMT-associated genes in ARID1A mutant and wildtype cells. E-cadherin, a member of the cadherins superfamily (transmembrane glycoproteins), mediates cell–cell movement by affecting the junction [4,36]. This classical marker of epithelial cells is important in epithelial cell adhesion and tissue architecture maintenance [34]. Moreover, the silence of ARID1A upregulated EMT markers N-cadherin and vimentin [37], so the levels of N-cadherin and vimentin were also measured. Compared to the ARID1A-wild-type cell line, the ARID1A-mutant cell line showed around a 6-fold increase of N-cadherin and a 12-fold increase of vimentin (Figure 3).

**Figure 4:**
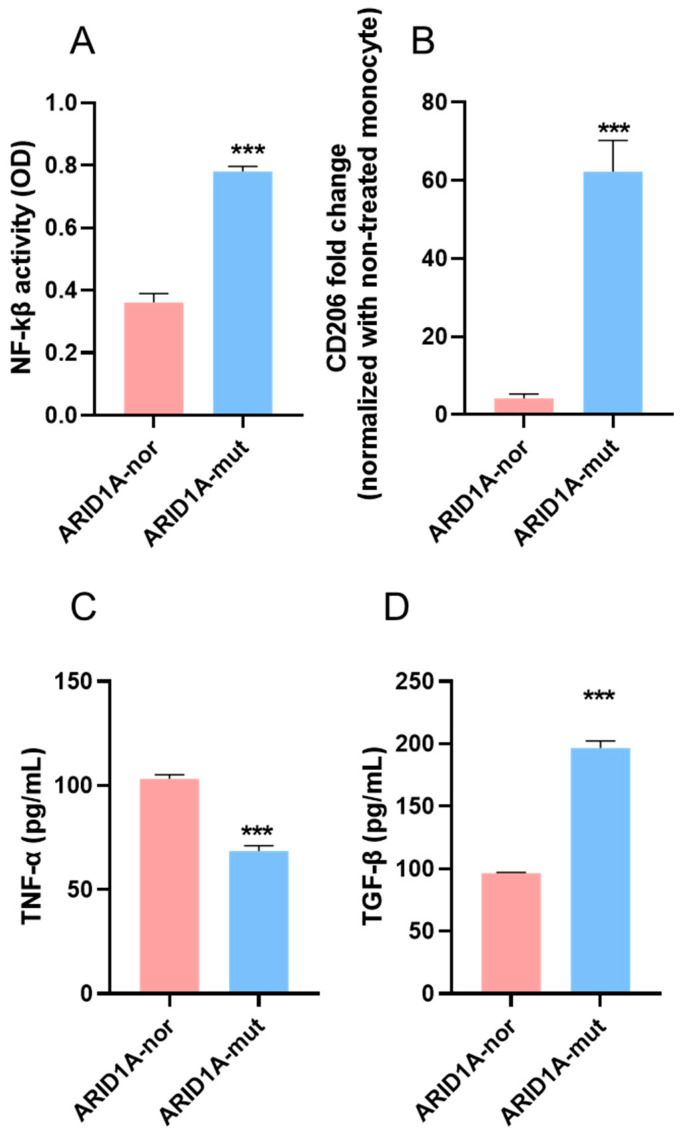
(**A**) Activity of NF-kβ in ARID1A wildtype (ARID1A-WT) and ARID1A-mutant (ARID1A-MT) cell lines, respectively. (**B**) Gene expressions of CD206 in macrophage co-cultured with the ARID1A-WT and ARID1A-mutant (ARID1A-MT) cell lines, respectively. (**C**) TGF-β levels of supernatants in the co-culture system. (**D**) TNF-α levels in supernatants in the co-culture system. (Data are presented as the mean ± SD, *n* = 3. ***, *p* < 0.001, unpaired *t*-test).

It was reported that the ARID1A-mutant cancer cell possessed aberrant metabolic activity, and the ARID1A mutant cell line showed a gain of nuclear factor-κB (NF-κB) activation [38]. This resulted in the promotion of cancer cell proliferation [16,39]. The higher NF-κB activity in the ARID1A-mutant cell line was found (Figure 4A). ARID1A-mutated cancers showed higher expression of PD-L1 than ARID1A-wildtype cancers, causing impaired tumor immunity [40,41].

Our studies demonstrated that the mutant cancer cell line results in a more immunosuppressive microenvironment (TIME) characterized by increased proliferation of cancer cells and other detrimental components, including M2TAM and TGF-β. However, we found that the combination of DQ-Lipo/Cu effectively decreased the levels of these harmful factors in the TIME. Furthermore, we observed that DQ-Lipo/Cu induced immunogenic cell death (ICD). The dying cancer cells displaying ICD could function as an in situ vaccine and activate the adaptive immune response, with or without the need for additional adjuvants. Given these promising findings, we believe that combining DQ-Lipo/Cu with other immuno-oncology therapeutics may be a highly effective treatment approach for cancer.

To know the impact of ARID1A-WT and ARID1A-MT on the monocyte phenotype, we measured the CD206 expression of monocyte in a co-culture transwell system comprised of cancer cells and monocyte. It showed that the CD206 level was higher in the ARID1A-MT co-culture group compared to the ARID1A-WT group (Figure 4B).

The trend was further verified by cytokine secretion (TNF-α and TGF-β). Tumor necrosis factor α (TNF-α) was identified as a cytokine that can cause cancer cell proliferation inhibition [42]. The TNF-α concentration in the ARID1A-WT co-culture group was higher than in the ARID1A-MT co-culture group, indicating and confirming the impaired immunity in the ARID1A-MT co-culture setting (Figure 4C). In contrast, TGF-β normally shows tumor-promoting activity and is produced by M2TAM [16,43]. In our study, we found the ARID1A-MT co-culture group had higher TGF-β release, suggesting the immunosuppressive microenvironment (Figure 4D).

### 3.4. In Vitro Antitumor Activity of Liposomes against ARID1A-Mutant Cancer Cell

In the assays mentioned above, we found the elevated ROS levels in the ARID1A-mutant cell line and DQ-Lipo/Cu exert more potential in inhibiting the ARID1A-mutant cell line. To confirm these findings and to know the efficacy of other components of liposomes. We further measured the ROS production in cells treated with the vehicle, CuCl_2_, DQ-Lipo, and DQ-Lipo/Cu, respectively.

**Figure 5:**
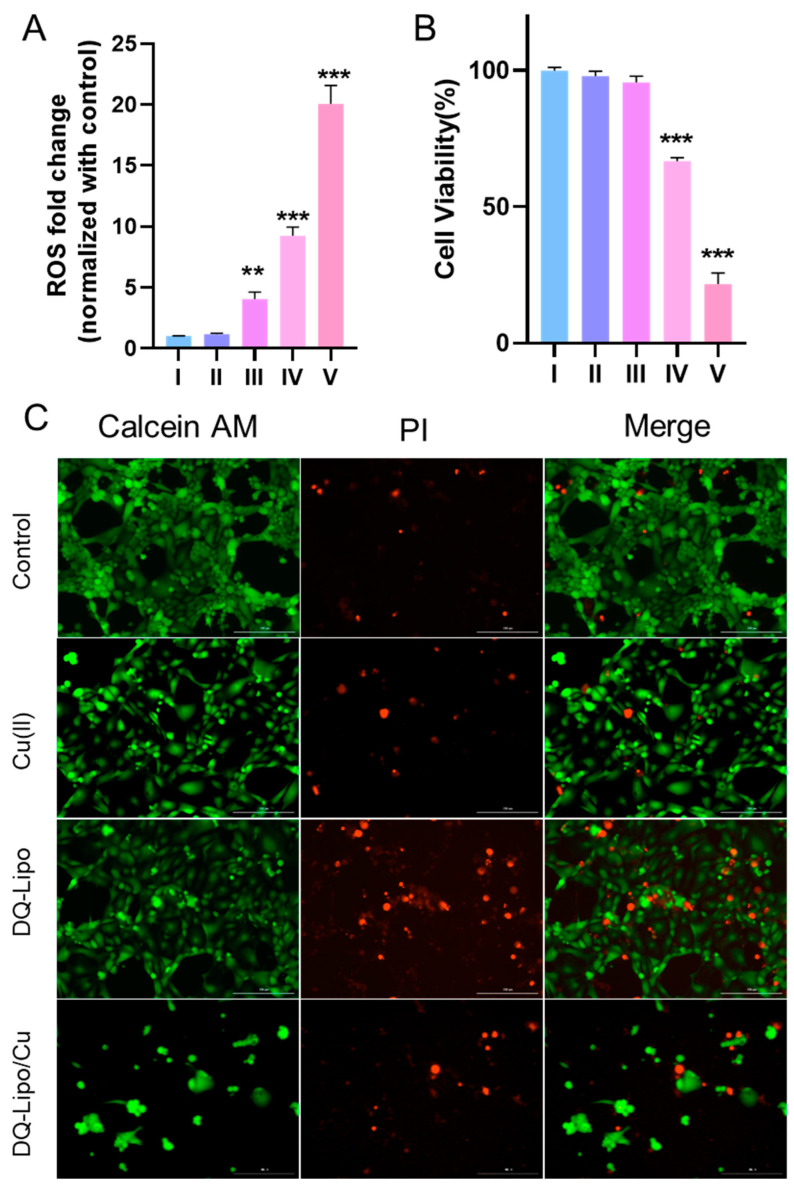
Effects of DQ-lipo/Cu treatments on ARID1A-mutant cell lines. (**A**) ROS generation in the ARID1A-mutant cell line with different exposures. (**B**) Cell viability of the ARID1A-mutant cell line after 48 h drug exposure. (Group setting: I Control group, II vehicle, III Cu, IV DQ-Lipo, V DQ-Lipo/Cu; **, *p* < 0.01, ***, *p* < 0.001, one-way ANOVA.) (**C**) The AM-PI staining in ARID1A-mutant cell line after 48 h exposure with or without treatments. Scale bar: 200 μm.

As shown in Figure 5A, because of the Fenton-like reaction, the copper also resulted in a minor ROS production. DQ-Lipo also increased the ROS levels. The DQ-Lipo/Cu group showed the most potent ROS increase, attributed to the chelation process being beneficial for ROS. The cell viability results shown in Figure 5B indicate the most significant inhibition effects of DQ-Lipo/Cu, followed by the single DQ-Lipo. However, the cell viability of the single Cu(II) group in this group did not show much change despite the slight increase in ROS. The level of ROS induced by copper was still not high enough, indicating the significance of DQ use. The Calcein AM/PI staining further confirmed this trend, with a greater PI signal indicating that the death cells suggested the much more potent efficacy of DQ-Lipo/Cu compared to other groups (Figure 5C).

**Figure 6:**
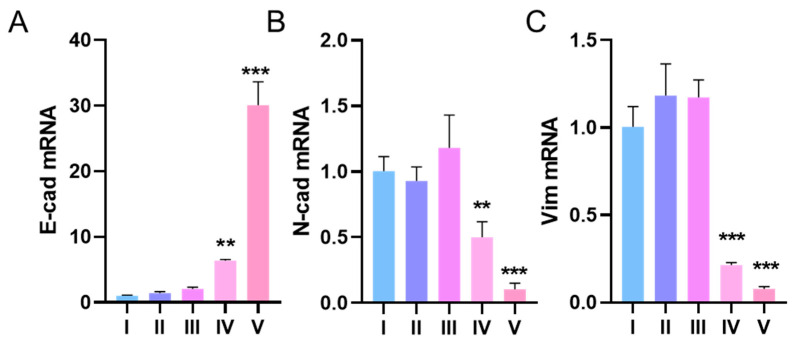
Effects of DQ-liposomes treatment on EMT biomarkers. (**A**) The change of E-cadherin in ARID1A-mutant cancer cells treated with different formulations for 24 h. (**B**) The change of N-cadherin in ARID1A-mutant cancer cells treated with different formulations for 24 h. (**C**) The change of Vim in ARID1A-mutant cancer cells treated with different formulations for 24 h. Data are presented as the mean ± SD, *n* = 3, ** *p* < 0.01, *** *p* < 0.001, one-way ANOVA. (Group setting: I. Control group, II vehicle, III, Cu IV. DQ-Lipo, V. DQ-Lipo/Cu).

E-cadherin expression was downregulated in the ARID1A mutation cell line. The decreased E-cadherin increased cell motility and promoted tumor metastasis [44]. We found the absence of ARID1A decreased E-cadherin and elevated N-cadherin and Vimentin (Figure 3). To learn the roles of DQ-based therapy towards the EMT markers, we used the qPCR to measure the gene expression of these markers. As indicated in Figure 6A, the E-cadherin was partially increased by the DQ-Lipo treatment, and the effects were much more potent in the DQ-Lipo/Cu group. Conversely, compared to the control group (I), the N-cadherin and Vimentin were significantly inhibited in the DQ-Lipo (IV) and DQ-Lipo/Cu group(V) (Figure 6B,C).

### 3.5. Effects of DQ-Lipo/Cu on Improvement of Cold TME

**Figure 7:**
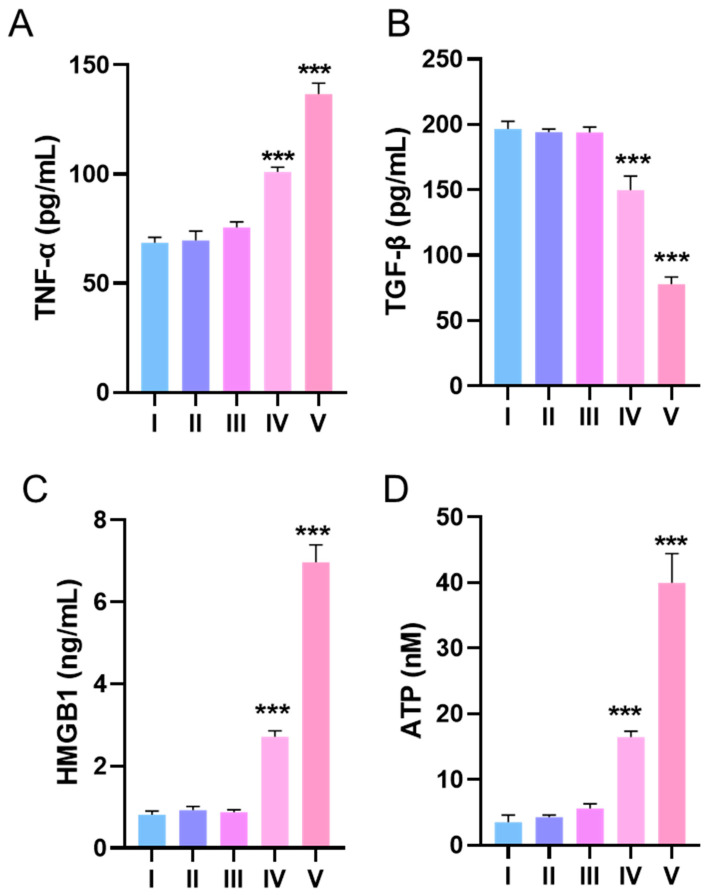
(**A**) Effects of different treatments on TNF-a levels in a co-culture system. (**B**) The effects of different treatments on TGF-b in a co-culture system. Data are presented as the mean ± SD, *n* = 3, *** *p* < 0.001. one-way ANOVA. (**C**) HMGB1 release in ARID1A-mutant cancer cells treated with different formulations for 24 h. (**D**) ATP release in ARID1A-mutant cancer cells treated with different formulations for 24 h. (Group setting: I Control group, II vehicle, III Cu, IV DQ-Lipo, V DQ-Lipo/Cu).

The cold microenvironment indicated a decrease in tumor-inhibitory factors but an increase in tumor-promoting factors [45]. Tumor necrosis factor-α (TNFα) was a positive factor in the cancer microenvironment showing anti-cancer efficacy [46]. The decreased TNF-α suggested the immune suppressive microenvironment induced by the ARID1A-MT co-culture. In contrast, treating DQ-Lipo and DQ-Lipo/Cu increased the TNF-α (Figure 7A). Oppositely, TGF-β promoted cancer cell proliferation and invasion [47]. In our study, we found that the ARID1A-MT co-culture with monocytes resulted in a higher release of TGF-β. However, our drug treatment increased the levels of TGF-β (Figure 7B). Different studies have shown that immunogenic cell death (ICD) induces biomarkers, such as ATP release and HMGB1 release [12]. Treated cancer cells act as “in situ tumor vaccines”; these vaccines convert a “cold” tumor immune suppressive microenvironment (TIME) to a “hot” one [48]. As shown in Figure 7D, extracellular ATP was increased in the DQ-Lipo and DQ-Lipo/Cu groups. Released ATP treated with therapeutics, such as DQ-Lipo and DQ-Lipo/Cu in the present study, could function as a short-range “find-me” signal, triggering an immune response. HMGB1 is another biomarker of ICD [16]. The released extracellular HMGB1 is crucial for the function of dendritic cells (antigen presentation). The activated immune response may also be responsible for the EMT regulation performance of DQ-Lipo/Cu.

## 4. Conclusions

In this study, we find a negative correlation between ARID1A expression and ROS levels in ovarian clear cancer cells. Increased ROS levels result in an abnormal redox balance in ARID1A-mutant OCCC. The combination of DQ-Lipo/Cu further disrupts this balance, resulting in cancer cell death and inhibition of EMT. Furthermore, DQ-Lipo/Cu induces ICD. The synergistic effects of DQ-Lipo/Cu in inhibiting cancer proliferation and associated EMT behavior make it a highly effective treatment for ovarian cancer. Our anti-cancer mechanism study strongly suggests that DQ-Lipo/Cu is a promising nanomedicine for controlling OCCC.

## Data Availability

Not applicable.

## Supplementary Materials

The following supporting information can be downloaded at: https://www.mdpi.com/article/10.3390/biom13050744/s1. Figure S1. The primers for qPCR; Figure S2. The ESI-MS spectrum of DQ (366.17 m/z); Figure S3. The levels of basal ROS ARID1A wildtype (ARID1A-WT) and ARID1A-mutant (ARID1A-MT) cell lines using DCFH-DA probe; Figure S4. The expression of ARID1A in mutant and wildtype cell line using western blot.
