# Supplementary material for: Liposomal DQ in Combination with Copper Inhibits ARID1A Mutant Ovarian Cancer Growth
Source: Biomolecules. 2023 Apr 25;13(5):744. doi: 10.3390/biom13050744 (PMC10216151; doi:10.3390/biom13050744)
Supplement: Supplementary file 1 [file biomolecules-13-00744-s001.zip › biomolecules-2220084-supplementary.pdf]

Name	Forward Sequence (5'-3')	Reverse Sequence (3'-5')
E-cadherin	CGGGAATGCAGTTGAGGATC	AGGATGGTGTAAGCGATGGC
Vimentin	CCTTGAACGCAAAGTGGAATC	GACATGCTGTTCCTGAATCTGAG
N-cadherin	CCTCCAGAGTTTACTGCCATGAC	GTAGGATCTCCGCCACTGATTC
CD206	AGCCAACACCAGCTCCTCAAGA	CAAAACGCTCGCGCATTGTCCA
Actin	CACCATTGGCAATGAGCGGTTC	AGGTCTTTGCGGATGTCCACGT

Figure S1. The primers for qPCR.

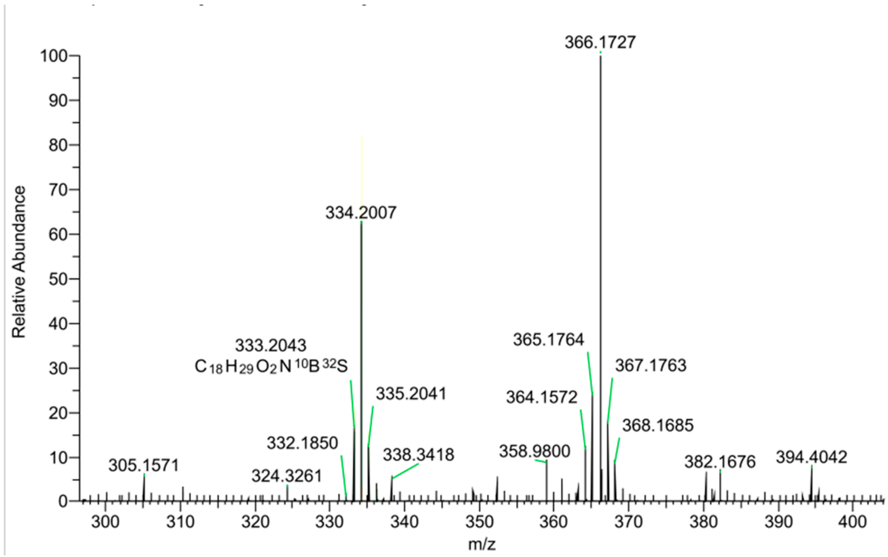

Figure S2. The ESI-MS spectrum of DQ (366.17 m/z).

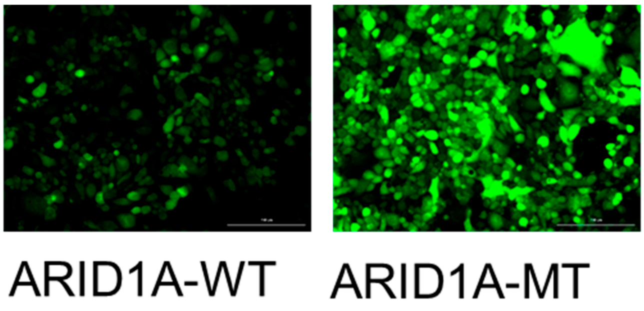

Figure S3. The levels of basal ROS ARID1A wildtype (ARID1A-WT) and ARID1A-mutant (ARID1A-MT) cell lines using DCFH-DA probe. Scale bar:200 um.

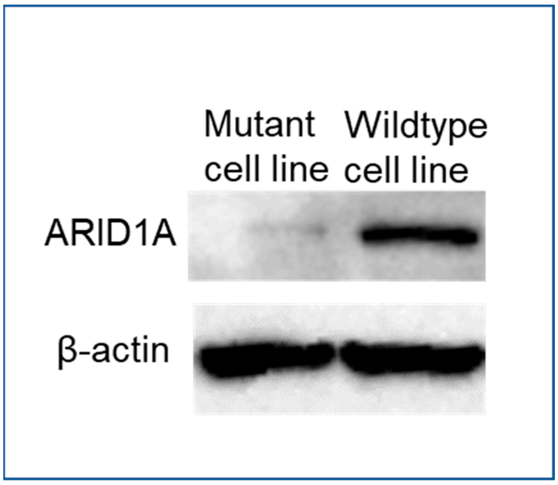

Figure S4. The expression of ARID1A in mutant and wildtype cell line using western blot.
